# Autovaccine-Based Immunotherapy: A Promising Approach for Male Recurrent Urinary Tract Infections

**DOI:** 10.3390/life14010111

**Published:** 2024-01-10

**Authors:** Alexandru Ciudin, Bernat Padulles, Razvan Popescu, Pasqualino Manasia

**Affiliations:** 1 Urology Department, Hospital Universitari de Mollet, 08100 Barcelona, Spain; b.padulles@fsm.cat (B.P.); p.manasia@fsm.cat (P.M.); 2 Urology Department, Spitalul Clinic “Prof. Dr. Th. Burghele”, 061344 Bucuresti, Romania; razvan-ionut.popescu@drd.umfcd.ro

**Keywords:** recurrent urinary tract infections (UTIs), autovaccine, immunotherapy, quality of life (QoL), incidence rate, IPSS

## Abstract

Background: Recurrent Urinary Tract Infections (UTIs) in men range from 0.9 to 2.4/1000 individuals in younger men to 7.7/1000 in those over 85, significantly impacting their quality of life. Preventive strategies include autovaccines, but limited evidence exists for males. Methods: A prospective monocentric, open-label observational study was conducted from August 2018 to August 2021, with follow-up until August 2023 including patients with recurrent UTIs treated with immunotherapy. We evaluated the incidence rate of UTIs per year, the incidence rate of episodes after two or three rounds of the autovaccine, and quality of life measured with the IPSS-QoL questionnaire. Results: A total of 49 patients fulfilled inclusion criteria. The mean age was 72 years (±15), and the median 61. The evolution of UTIs number of episodes after the autovaccine rounds: −37.74% for the first round from 5.3 to 3.3; −33.33% for the second round from 3.3 to 2.2; −45.45% for the third round from 2.2 to 1.2. The mean IPSS score improved from 10.69 to 7.27 after the treatment (32%). The mean QoL subscore enhancement was from 4.22 to 1.92 (54%). With a mean follow-up of 3 years, only nine patients required retreatment. Conclusion: Autovaccine treatment significantly reduced the number of UTI episodes, with a cumulative effect observed after multiple rounds of treatment, demonstrating an enhancement in QoL and with sustained effectiveness and a low need for retreatment.

## 1. Introduction

Urinary Tract Infections (UTIs) are a common health issue affecting both men and women. However, recurrent UTIs in men present unique challenges and are less understood compared to their female counterparts. Recurrent UTIs are defined as the presence of two or more symptomatic infections within a six-month period or three or more infections within a year. Although recurrent UTIs are more common in women, around 20% of all diagnosed UTI cases occur in men. Despite the lower incidence, approximately 13–14% of men will experience at least one UTI in their lifetime, compared to around 50% of women [1,2].

The frequency of recurrent UTIs in males varies with age. In men under 55 years old, there is an estimated 0.9 to 2.4 cases of UTIs per 1000 individuals. However, in men over 85 years old, this number increases to around 7.7 cases per 1000 individuals [3,4,5]. A study conducted in male veterans revealed that 1.46% reported having a UTI in the past year, and among these cases, nearly 15% were recurrences [6].

One complicating factor in managing UTIs in males is the interrelation with chronic bacterial prostatitis (CBP). Bacterial prostatitis should be considered a variant of UTIs, as patients with UTI and bacterial prostatitis share similar symptoms. The bacteria responsible for the infection are largely the same. CBP has been identified as the most common cause of recurrent UTIs in young to middle-aged men. Around 90% of men with febrile UTIs and 50% of men with recurrent UTIs show some degree of prostate involvement [2].

UTIs and CBP can present overlapping symptoms, further complicating diagnosis and treatment. Symptoms include pain during urination, urinary urgency and frequency, abdominal or lower back pain, among others. In the case of CBP, additional symptoms include perineal discomfort, pain during ejaculation, and general discomfort. This overlap underscores the need for effective and specific therapeutic approaches [2].

Animal research has indicated that men may exhibit a weaker immune response to UTIs, possibly due to the role of androgenic hormones. Both men and women with higher testosterone levels are more likely to suffer from chronic long-lasting UTIs [7,8].

Immunoprophylaxis in the form of autovaccines has been used as a strategy to prevent recurrent urinary tract infections (UTIs) in some patients and currently has the highest recommendation grade while being cost efficient [2,9,10]. Nevertheless, the recommendations are the same for both genders, even if most of the evidence was obtained in women. There is limited evidence regarding the clear indication of autovaccine use in the prophylactic treatment of recurrent UTIs in males [2,11,12,13].

As an alternative in some cases, long-term prophylactic antibiotics can be prescribed to prevent future infections. However, prolonged use of antibiotics can increase the risk of antibiotic resistance and other side effects, such as dysbiosis. Suppressive antibiotic treatment is recommended by the EAU Guidelines when non-antibiotic measures have failed [2].

Vaccines have been used for a long time to prevent repeated urinary tract infections. Initially, the vaccines were combinations of collections of bacterial strains, at first only of Escherichia coli strains (OM-89), but later evolving to combined formulas that include a fixed percentage of bacteria or even being able to prescribe flexible combinations according to the patients’ previous urine cultures. Autovaccines came as the final evolution of this process, using not collection bacteria but the patient’s own bacteria, being able to further personalize the treatment of repetitive UTIs in each case [2].

The mechanism of action of vaccines and autovaccines for recurrent UTIs is to stimulate the production of antibodies in mucosa and the activation of T cells and macrophages. Due to this mechanism, the oral route is usually used for administration, considering that it favors the stimulation of the mucous membranes. There is no evidence that relates the amount of antibodies in the blood to the antibodies in the mucosa, therefore tests to assess the amount of plasma antibodies such as agglutination and hemagglutination would not be useful in our case [14,15].

The concept of using autovaccines for the prophylaxis of recurrent urinary tract infections (UTIs) does not necessarily require synchronization with the onset of infections. Research has indicated that the use of polybacterial vaccines can be administered with the aim of reducing the number of recurrent infections. These vaccines are given to stimulate the immune system to prevent future infections. In various studies, patients were administered vaccines for a period of 3 months, and the primary outcome was the reduction in the number of infectious episodes. This approach aligns with the concept of using vaccines prophylactically, rather than in response to an active infection [16,17].

In addition to reducing the number of UTIs, vaccines have the potential to also reduce the costs associated with repeat infections. In a study that compared the use of vaccines with the use of prophylactic antibiotics, it was demonstrated that per patient consumption of antibiotics, consultations, emergency room visits, and complementary exams significantly decreased, resulting in a reduction in healthcare expenditure per patient/year from mean (SD) 1001.1 (655.0) to 497.1 (444.4) EUR [10].

On these bases, the hypothesis of this study is that immunotherapy using autovaccination will be effective in significantly reducing the recurrence rate of UTIs in a male population diagnosed with recurrent UTIs. It is expected that the application of autovaccination will lead to a measurable reduction in the number of UTI episodes over a specific period of follow-up.

Additionally, it is hypothesized that multiple rounds of autovaccine treatment will result in a cumulative effect, further enhancing the reduction in UTI recurrence rates. This cumulative effect is anticipated to reflect an adaptive strengthening of the immune response over time, leading to improved resistance against UTI-causing pathogens.

For this purpose, we designed the present study with the following objectives:

### 1.1. Primary Objective

To evaluate the efficacy of immunotherapy using autovaccination in reducing the recurrence rate of Urinary Tract Infections (UTIs) in a male population diagnosed with recurrent UTIs.

### 1.2. Secondary Objectives

1. To assess the cumulative effect of multiple rounds of autovaccine treatment on the reduction of UTI recurrence rates in males with recurrent UTIs.
2. To compare the rate of decrease in UTIs after each round of autovaccine treatment, utilizing the rate of change formula, and to analyze the presence of an accumulative biological response over time.

## 2. Materials and Methods

A prospective monocentric, open label observational study was conducted at our site since August 2018, enrolling patients until August 2021 and with follow-up until August 2023, including male patients diagnosed with recurrent UTIs treated with immunotherapy using autovaccination.

### 2.1. Inclusion Criteria

To be included in the study, patients had to meet the following additional criteria:

1. Male gender.
2. Age > 18 years.
3. No previous immunotherapy treatment for UTIs.
4. Follow-up of at least one year from the start of treatment.

### 2.2. Exclusion Criteria

1. Previous prostate or bladder surgery.
2. Diuretic treatment.
3. Chronic kidney disease.
4. Additional pathologies that could be the cause of symptoms similar to UTI such as bladder lithiasis or bladder tumor, vesicoureteral reflux, renal or ureteral stones, and urinary tuberculosis.
5. Concomitant suppressive antibiotic treatment.

### 2.3. Treatment Procedure

Patients selected for the study received treatment through immunotherapy using an autovaccine (Probelte Pharma, Murcia, Spain). The autovaccine administration process involved the following steps:

1. Patients provided urine and semen samples in the clinic.
2. Smears of both samples were prepared and sent through the standard process to the autovaccine producer.
3. The patients’ own exudates were cultured, killed and lysed. The final product was in the form of a sublingual solution, containing 2000 million germs per milliliter. No adjuvants were added.

Once the autovaccine was received, the treatment was initiated progressively. Patients started with an initial dose of one drop and gradually increased the dose to reach approximately 5 sublingual drops daily, maintaining this regimen for about three months.

### 2.4. Evaluation and Follow-Up

Patient evaluation was performed six months after completing the autovaccine treatment, with a check including urine analysis, semen analysis and abdominal ultrasound. The criteria for considering treatment successful were asymptomatic patients in relation to UTIs or up to one UTI episode every six months. In cases where the presence of recurrent UTI criteria persisted after the initial treatment, the administration of a new round of autovaccine was considered. Up to three autovaccine rounds were allowed for each patient.

### 2.5. Outcomes

The primary outcome was the incidence rate of infectious episodes per year, calculated from the total number of episodes after at least 6 months of follow up after the treatment. As secondary outcomes we evaluated the incidence rate of episodes after 2 or 3 rounds of autovaccine, the evolution of International Prostate Symptoms Score, and the quality of life measured with the IPSS-QoL questionnaire.

### 2.6. Patient Data

1. Patient’s age.
2. Number of autovaccines.
3. International Prostate Symptom Score (IPSS).
4. Prostate volume obtained from the ultrasound result.
5. Number of urinary tract infection (UTI) episodes. The diagnosis of a UTIs episode is based on clinical evidence, i.e., the presence of dysuria, frequency +/− hematuria, fever.
6. Medical history: Includes information about other preexisting medical conditions that could impact treatment efficacy and autovaccine response, such as diabetes, heart diseases, or other comorbidities.
7. Urine and semen analysis results: Specific details of urine and semen analyses conducted on patients, including presence of bacteria, leukocytes, epithelial cells, and other relevant markers.
8. Previous treatment history: Whether patients had received any prior treatment for UTIs, such as antibiotics or alternative therapies.
9. Health-related quality of life: Data evaluating patients’ quality of life before and after treatment, such as surveys or health-related quality of life scales.

### 2.7. Statistical Analysis

The data obtained from this study were subjected to a comprehensive statistical analysis to assess the effectiveness of immunotherapy using autovaccination in reducing the recurrence rate of Urinary Tract Infections (UTIs) and associated symptoms in the male population with recurrent UTIs.

Descriptive statistical methods, including means, medians, and standard deviations, were employed to summarize the results of the study. Success rates were calculated based on the defined criteria for treatment success, which included being asymptomatic in relation to UTIs and experiencing up to one UTI episode every six months. Recurrence rates were determined by tracking the number of UTI episodes over the follow-up period.

For the analysis of the cumulative effect of multiple autovaccine rounds, the rate of change formula was utilized. This formula expressed the percentage change in the number of UTIs after each round of treatment compared to the initial value. These calculations aimed to elucidate the presence of a cumulative biological response to autovaccination over time. To calculate the rate of decrease in UTIs, we can use the rate of change formula, expressed as: Rate of Change (%) = ((Final Value − Initial Value)/Initial Value) × 100.

The comparison between the number of autovaccine rounds required for elderly patients (aged over 61 years) and younger patients (aged under or equal to 61 years) was analyzed using the Mann–Whitney U test, also known as the Wilcoxon rank–sum test.

The study was not statistically powered given the exploratory design and the absence of previous literature on the topic.

## 3. Results

We evaluated 667 patients diagnosed with recurrent UTIs and treated with immunotherapy using autovaccines. A total of 49 patients fulfilled the criteria for being included in the study. They had a mean age of 72 years (±15), and a median of 61 years. Before receiving immunotherapy treatment through an autovaccine, patients had an average of 5.3 urinary tract infections (UTIs) per year. After treatment, a marked decrease in the number of UTIs was observed, reducing to an average of 1.2 infections per year. Furthermore, the average number of autovaccine rounds needed was 1.8 (±0.8).

Of the 49 patients included in the study, 26 needed only one round of autovaccine to meet the success criteria. The remaining 23 out of 49 (46.9%) needed more than one round of autovaccine, as they continued to meet the criteria for autovaccine treatment despite improvement.

The rate of change of UTIs number of episodes after the autovaccine rounds: −37.74% for the first round from 5.3 to 3.3; −33.33% for the second round from 3.3 to 2.2; −45.45% for the third round from 2.2 to 1.2 (Table 1). These calculations represent the rates of decrease in the number of UTIs episodes after each round of autovaccine compared to the initial value. Negative rates indicate a decrease in the number of UTIs episodes, supporting the observation of a cumulative treatment effect.

All patients had a postvoid residual volume of less than 50 cm^3^. In uroflowmetry, the Qmax was 12.5 mL/s (median 14.3 mL/s, range 7.1 mL/s to 20.1 mL/s).

IPSS score improved after the treatment, from a mean IPSS of 10.69 to a mean IPSS 7.27 after the treatment, a 3.42 points difference (32%) (Table 2). Overall, the data suggests that younger patients and those with a smaller prostatic volume tend to experience more substantial improvement in IPSS scores following treatment. This trend is particularly evident and significant from a statistical point of view in the 40 and 50 year age groups. As patients age and prostatic volume increases, the magnitude of IPSS improvement tends to decrease, with a more noticeable decline in the older age groups.

The Quality of Life (QoL) subscore derived from IPSS improvement exhibited positive outcomes. Notably, all patients demonstrated an enhancement in their QoL subscore from a mean QoL of 4.22 to 1.92 (2.3 points/54% improvement) (Table 3). Interestingly, the younger patient group and those with smaller prostatic volumes experienced the most pronounced improvements, aligning with the observed decrease in IPSS scores.

In all patients, urine and semen samples were collected. All identified bacteria were considered candidates for inclusion in the autovaccine. Out of the 79 vaccines administered to the 49 patients, only in 9 cases were 3 bacteria identified simultaneously: in 3 cases, Escherichia coli, Klebsiella pneumoniae, and Pseudomonas aeruginosa; and in 5 cases, Escherichia coli, Klebsiella pneumoniae, and Pseudomonas aeruginosa. In the remaining 70 autovaccines, one or two bacteria were identified (Table 4).

Among 23 patients requiring multiple autovaccine rounds, 11 showed consistent bacterial reoccurrence as the same bacteria was identified in every control, while 12 displayed varied cultures across cycles. Among patients with one bacterium (17), fewer rounds (avg. 1.61) were needed compared to those with multiple bacteria (22, avg. 1.78, *p* = 0.12).

Elderly patients (more than 61 years, the median of age) require less autovaccine rounds—1.53 than the younger ones—1.83, *p* = 0.04.

The mean follow-up was 3 years, with a median of 2.4 years. Once the success criteria were met, a mere nine patients required retreatment, demonstrating the sustained effectiveness of the autovaccine therapy.

No adverse effects that could be related to the use of the autovaccine were reported.

## 4. Discussion

This research represents a milestone in the field of recurrent UTIs in the male population, focusing on an issue that has often been overlooked in previous studies. While some previous studies included men in their cohorts [18,19], the lack of specific analysis of this population limited the understanding of their response to treatment. This study marks a step forward by exclusively focusing on males with recurrent UTIs, thereby providing a deeper insight into this issue within this demographic group.

The results obtained in this study are in line with global trends observed in the efficacy of autovaccines in other conditions [12,13,14,19,20,21]. Immunotherapy using autovaccines has proven to be highly effective in significantly reducing the recurrence rate of UTIs from 5.3 to 1.2 episodes per year in the studied male patients. This once again confirms the promising ability of autovaccines to modulate the immune response and address UTI recurrence.

UTIs are primarily diagnosed based on clinical symptoms and confirmed with urine cultures. Systemic parameters, such as blood tests, are not commonly used in either the diagnosis of UTIs or the evaluation of the effectiveness of preventive treatments. Symptoms are the most important criterion because in the case of patients with asymptomatic bacteriuria, no type of treatment is indicated except in the case of immunosuppressed or pregnant patients [2]. That is why we use the number of infections as the main criterion for evaluating the success of preventive treatment.

A key observation lies in the presence of a cumulative effect in reducing UTIs as patients completed multiple rounds of autovaccines. Patients who, after the first round, only showed improvement but still met the criteria for recurrent UTIs, benefited from treatment with additional rounds of autovaccines, consistent with the data described in other studies [18]. This pattern suggests an accumulative biological response that could be related to adaptation and strengthening of the immune system over time. This observation aligns with other treatments involving immunotherapy and reinforces the importance of considering multiple treatment rounds to achieve optimal and sustainable results.

In the subgroup of patients requiring more than one round of autovaccine administration, we observed intriguing patterns in bacterial recurrence and variability across treatment cycles. Out of the 23 patients who necessitated multiple rounds of autovaccination, a noteworthy distinction emerged: 11 patients exhibited a persistent recurrence of the same bacterial strain, signifying a remarkable resilience of that particular pathogen or a very low activation of the immune system for that strain. Conversely, in 12 patients, the results of microbial cultures were different between successive autovaccine administrations, suggesting a good response of the immune system to the initial bacteria but also supporting the idea that as intrinsic immunity controlled the activity of one bacterium, others could multiply and replace it.

Furthermore, when evaluating the subgroups of patients harboring a single bacterial strain and those with multiple bacterial species, we uncovered compelling disparities in their autovaccine response. Specifically, the subset of patients with a solitary bacterial species, 17 in total, necessitated fewer rounds of autovaccine administration, with an average of 1.61 rounds, compared to their counterparts with a polymicrobial profile, who required an average of 1.78 rounds. Despite a discernible difference in favor of the single-bacterial cohort, statistical analysis (*p* = 0.12) indicated that the variance did not achieve significance at the predetermined threshold.

These findings collectively underscore the intricate nuances that underlie the efficacy of autovaccine therapy in distinct patient cohorts. While the data suggest a potential advantage for patients with a solitary bacterial strain in terms of treatment duration, the lack of statistical significance prompts a call for further investigation to elucidate the underlying mechanisms driving these observations. The interplay between bacterial diversity, individual immune responses, and the evolving microbiome landscape remains an enticing area for future research, with the potential to enhance our understanding of personalized therapeutic strategies for recurrent urinary tract infections.

Uro-Vaxom (OM-89^®^) and Urovac have proven to be effective and safe treatments for the prophylaxis of recurrent urinary tract infections. Uro-Vaxom, in particular, has shown a significant reduction in the recurrence rate of infections, especially 3 months after treatment. A retrospective study revealed a decrease in the average number of urinary tract infections in the year following the start of Uro-Vaxom compared to the year before its administration [22]. A systematic review including Uro-Vaxom and Urovac indicated that both can reduce the recurrence of urinary tract infections [14]. On the other hand, MV-140, a sublingual treatment, has shown similar effectiveness in reducing recurrent urinary tract infections, offering a promising non-antibiotic alternative [19]. The choice between these treatments should be based on the individual circumstances of the patient and the clinical consideration of the doctor.

Since 2018, in Spain, regulations limit the use of treatments for recurrent urinary tract infections to autovaccines, which excludes the use of options like MV-140 or Urovaxom. This regulation emphasizes the preference for treatments based on autovaccines, which are customized according to the specific pathogens of the patient. This approach aims to improve efficacy and reduce the risks associated with more generalized treatments. Therefore, although Uro-Vaxom and MV-140 have proven effective in studies, their use in Spain is restricted in favor of autovaccines. 

When considering the autovaccination regimen in relation to age, we saw that elderly patients, defined here as those aged over 61 years (the median age of the study cohort), demonstrated a notably reduced requirement for autovaccine rounds compared to their younger counterparts (with a median of 1.53 rounds for the elderly versus 1.83 rounds for the younger group). This intriguing finding introduces a multifaceted perspective on how age influences the therapeutic response to autovaccination in the context of recurrent urinary tract infections (UTIs).

Delving into this age-specific phenomenon sparks inquiries into the potential biological and immunological factors that contribute to the divergent autovaccination requirements observed between elderly and younger patients. One plausible interpretation is that the ageing immune system, which often exhibits characteristics of immunosenescence, may respond more promptly and robustly to the autovaccine. It is conceivable that the cumulative effects of a lifetime of immune challenges and exposures may have primed the immune response in elderly patients, leading to a more efficient and effective reaction to the autovaccination [23,24].

Once the success criteria were met, a mere nine patients required retreatment, demonstrating the sustained effectiveness of the autovaccine therapy. This low need for retreatment underscores the potential of this approach to provide lasting benefits and contribute to a reduced burden of recurrent urinary tract infections. The extended follow-up period provides valuable insights into the prolonged benefits of autovaccine therapy, further supporting its viability as a promising therapeutic intervention.

In contrast to the well-documented adverse effects associated with prolonged antibiotic courses reported in international literature [2,25,26,27], our study revealed a notable absence of adverse effects in patients undergoing multiple autovaccine rounds. This finding highlights a potential advantage of autovaccine therapy, suggesting a safer and more tolerable approach compared to traditional antibiotic regimens. The absence of adverse effects underscores the potential value of autovaccines as a promising therapeutic option for recurrent urinary tract infections, presenting a viable alternative with potentially reduced risks and improved patient outcomes. Further research is warranted to validate these findings and explore their long-term implications.

While recurrent UTIs are less common in males compared to females, their persistence can be more pronounced due to hormonal factors, such as the presence of testosterone [7,8]. The underlying pathophysiology in males appears to share similarities with that in females, supporting the extrapolation of the indication for immunotherapy treatment. The immune response could be influenced by similar hormonal factors in both sexes, suggesting that immunotherapy principles could be effectively applied in male populations as our study demonstrates.

This study also highlights the need to consider a gender-based approach in the research and treatment of recurrent UTIs. While current clinical guidelines do not differentiate by sex in terms of treatment, it is plausible that the perspective will evolve as research in this area increases. The present study lays the groundwork for future work that could influence recommendations and treatment guidelines, especially in the context of recurrent UTIs in men. Despite the significant contributions of this study, it is important to recognize some limitations. First, the single-center nature of the research may limit the generalizability of the findings. The study was conducted at a single site, which might not fully reflect the variability and diversity of patients with recurrent UTIs across different geographical and clinical settings. Second, the absence of a control group in our study design represents a significant limitation. Without a comparison group receiving a placebo or standard treatment, it is challenging to accurately determine the efficacy of autovaccine compared to other therapies. This limitation is further exacerbated by the observational nature of the study, which, while providing valuable data on real-world clinical practice, may be subject to inherent biases such as selection bias. Furthermore, although our sample was representative of the patients attending our center, the study population was relatively small. This raises questions about the statistical power and the ability to detect significant differences or generalize the findings to a broader population. Lastly, although we have followed up with patients over a considerable period, studies with longer follow-ups could provide a deeper understanding of the long-term efficacy and safety of autovaccine in the treatment of recurrent UTIs. Future studies could address these limitations through multicentric designs, the inclusion of a more robust control group, and a broader and more diverse study population. Despite these limitations, the current findings provide a strong foundation for future research and advancements in the understanding and treatment of recurrent UTIs in men.

The results of our study suggest a substantial reduction in UTI recurrence and highlight the relevance of considering multiple autovaccine rounds to harness a cumulative effect. While further research is required, this study represents a step forward in the understanding and management of recurrent UTIs in men, opening new therapeutic perspectives and contributing to the advancement of medical knowledge in this field.

## 5. Conclusions

Autovaccination proves to be a highly effective tool in the prophylaxis of UTIs in patients with chronic bacterial prostatitis, with a cumulative effect and potential to improve the quality of life. Its application represents a significant advancement in the treatment of this understudied male population.

## Figures and Tables

**Table 1:** The rate of change of UTIs number of episodes after the autovaccine rounds.

Autovaccine Round	Mean Number of UTIs	Decrease from Previous Round	*p* Value
First round	5.3	n/a	
Second round	3.3	−37.74%	0.03
Third round	2.2	−33.33%	0.03
After the 3rd round	1.2	−45.45%	0.01

**Table 2:** IPSS improvement stratified by age and prostate volume.

Age Group	Prostate Volume < 40 cm³	Number of Patients	Mean Prostate Volume (cm³) ± SD	Median Prostate Volume (cm³)	Mean Initial IPSS	Mean IPSS after Treatment	Mean IPSS Improvement	*p* Value
40–49	Yes	6	37.4 ± 2.3	37.8	8.6	4.4	48.3%	0.03
40–49	No	2	64.7 ± 3.9	65.0	9.3	5.6	40.1%	
50–59	Yes	5	39.5 ± 2.8	39.5	9.9	4.9	50.3%	0.03
50–59	No	3	65.7 ± 3.4	66.0	10.1	6.3	38.1%	
60–69	Yes	5	38.0 ± 2.5	37.5	11.0	7.8	29.1%	0.18
60–69	No	6	52.2 ± 1.9	52.5	10.7	7.6	28.9%	
70+	Yes	8	37.9 ± 1.6	38.0	10.5	6.9	34.3%	0.01
70+	No	14	43.8 ± 1.3	43.5	12.2	9.7	20.5%	

**Table 3:** QoL improvement stratified by age and prostate volume.

Age Group	Prostate Volume < 40 cm³	Number of Patients	Mean Prostate Volume (cm³) ± SD	Median Prostate Volume (cm³)	Mean Initial QoL	Mean QoL after Treatment	Mean QoL Improvement	*p* Value
40–49	Yes	6	38.2 ± 2.5	37.8	3.7	1.0	73%	0.021
40–49	No	2	45.9 ± 4.1	46.2	4.1	1.5	63%	0.032
50–59	Yes	5	36.5 ± 3.0	36.2	4.0	1.2	70%	0.014
50–59	No	3	43.8 ± 3.5	44.1	4.5	1.8	60%	0.027
60–69	Yes	5	32.1 ± 2.8	31.9	4.5	1.5	75%	0.009
60–69	No	6	40.6 ± 3.2	40.9	4.9	2.2	55%	0.023
70+	Yes	8	28.7 ± 2.0	28.5	4.8	1.8	62.5%	0.007
70+	No	14	37.2 ± 2.9	37.4	5.2	2.5	51.9%	0.031

**Table 4:** Distribution of diagnosed bacteria. The percentage distribution sums up to more than 100% due to the fact that several patients had more than one bacterium identified.

Bacterium	Percentage Distribution
*Escherichia coli*	35.9%
*Enterococcus faecalis*	25.6%
*Klebsiella pneumoniae*	22.2%
*Pseudomonas aeruginosa*	20.7%
*Proteus mirabilis*	12.9%
*Klebsiella oxytoca*	5.6%
*Enterobacter cloacae*	4.6%
*Serratia marcescens*	1.85%
*Candida albicans*	0.93%
*Pseudomonas* sp.	0.93%
*Staphylococcus* spp.	0.93%
*Staphylococcus epidermidis*	0.93%
*Citrobacter freundii*	0.93%
*Serratia* sp.	0.93%

## Data Availability

Data are not available due to the Spanish Organic Law on Data Protection.
